# Cognitive dynamics of verb-particle constructions: an eye-tracking study on phrasal verbs and verb-preposition combinations

**DOI:** 10.3389/fpsyg.2026.1676341

**Published:** 2026-02-16

**Authors:** Hassane Kissane, Konstantin Tziridis, Achim Schilling, Thomas Herbst, Patrick Krauss

**Affiliations:** 1 Chair of English Philology and Linguistics, University Erlangen-Nuremberg, Erlangen, Germany; 2 Cognitive Computational Neuroscience Group, Chair of Pattern Recognition, University Erlangen-Nuremberg, Erlangen, Germany; 3 Neuroscience Lab, University Hospital Erlangen, Erlangen, Germany; 4 University Hospital Mannheim, University Heidelberg, Heidelberg, Germany; 5 BGU, Ludwigshafen, Germany

**Keywords:** cognitive processing, eye-tracking studies, lexical access, linguistic processing, phrasal verbs, prepositional verbs, psycholinguistics, reading comprehension

## Abstract

This study investigates the cognitive processing of verb-particle constructions (VPCs) using eye-tracking data to explore how English native speakers process different types of the sequence noun phrase (NP)-verb-particle-NP during reading tasks. While previous research has focused on phrasal verbs, our study extends this examination to include patterns with prepositions, aiming to identify distinct cognitive engagement patterns and processing efficiencies associated with each. We employed the Provo Corpus to analyze eye movements while participants read sentences containing these constructions, focusing on metrics such as first fixation duration, gaze duration, go-past times, and total reading times. Our findings indicate similarities in the lexical verbs, and significant differences in particles, indicating how these two types of constructions are processed, with phrasal verbs sometimes processed more efficiently than the prepositional counterparts. This suggests that phrasal verbs might be more deeply entrenched in the linguistic repertoire of native speakers, possibly functioning as single lexical units. However, larger and more systematically controlled item sets are needed to test the generality of this interpretation. This research contributes to the understanding of complex structures processing and the cognitive mechanisms that support it, offering insights that could influence linguistic theory and language education.

## 1 Introduction

### 1.1 The linguistic problem: identifying verb-particle combinations

Combinations of verb and particle have always attracted a lot of attention in the analysis and the teaching of the English language. One indication of this is the great number of so-called phrasal verb dictionaries flooding the market of English learners’ dictionaries – a type of dictionary that does not exist in this form for other Germanic languages. Since articles, adjectives and, with the exception of the genitive, nouns show no case marking in present-day English, the relations between the elements of clauses have to be expressed in other ways. Apart from word order, it is prepositions that play an important part in making syntactic relations clear. This is certainly one of the reasons why combinations of verbs with “small” function words such as “to,” “in” or “about” have attracted a lot of attention, another one being that many such combinations can be analyzed as being “idiomatic,” i.e., as expressing a meaning that cannot easily be derived from its component parts—just think of “look after” in the sense of taking care of someone or something.

Despite (or because of) the importance of verb-particle combinations in present-day English, there is some terminological inconsistency in the use of the term phrasal verb and, indeed, there are various ways in which such combinations have been analyzed. While phrasal verb dictionaries tend to have a very wide concept of phrasal verbs, others use the term only for one type of verb-particle combination. For instance, one of the standard reference grammars of the English language, the Comprehensive Grammar of the English Language by Quirk et al. (1985) make a distinction between three main types, namely:

(i) prepositional verbs,^1^ in which the prepositional particle always precedes the noun phrase that these grammars treat as the direct object as in:

(1) a. I agree with Shawn. _TV-2010-Shawn_.

b. ?? I agree Shawn with.

(ii) phrasal verbs, in which the adverbial particle can precede or follow the noun phrase as in.

(2) a. … look up the word “semantics.” _TV-2010-Psych_.

b. You can look the word up. _TV-2008-Psych_.

(iii) phrasal-prepositional verbs,^2^ which are combinations of a verb and two particles (adverbial and prepositional):

(3) I look forward to the opportunity to get to know you better. _TV-2012-Psych_.

Not all linguistic models approach verb–preposition constructions in the same way. There is a major difference between the approach taken by Quirk et al. (1985) and that of the valency model, which arose from Tesnière’s dependency theory and has also been applied to English (Herbst et al., 2004; Herbst and Schüller, 2008; Herbst and Uhrig, 2019). Both Quirk et al. (1985, pp. 1156–1163) and the valency model recognize the noun phrase following the preposition as a prepositional object, but they differ in how they represent the structure of verb–preposition combinations. In Quirk et al.’s analysis, certain combinations (e.g., look after) are treated as prepositional verbs—lexical units that resemble transitive verbs. In contrast, the valency approach does not assume lexical unity between the verb and the preposition, but treats the prepositional phrase as a syntactic complement selected by the verb (cf. Huddleston and Pullum, 2002, p. 274).

What these different classifications have in common is that they capture different degrees of association between a verb and a particle. While structural classifications focus on syntactic criteria, usage-based approaches additionally consider factors such as frequency and meaning, which may influence the strength of association between a verb and its particle. For instance, corpus-based transitional probabilities (Jurafsky, 2003) indicate that some verb–particle sequences show strong co-occurrence patterns, suggesting that frequency and predictability may affect how such combinations are processed during reading.

The classification introduced earlier (i, ii, iii) describes formal syntactic types of verb-particle constructions as proposed in reference grammars such as Quirk et al. (1985). Table 1 reorganizes these constructions from a complementary perspective, grouping them along a gradient of verb-particle integration and lexical cohesion. The two classification schemes are therefore not competing but address different analytical goals: one focuses on formal syntactic properties, while the other highlights degrees of association that are particularly relevant for psycholinguistic processing.

**Table 1:** Types of verb-particle constructions organized in terms of structural properties and degrees of verb–particle integration (NP, noun phrase; PP, prepositional phrase).

Type	Description	Shiftability	Stress
A	verb + PP-adjunct *(*e.g.*, work in the park)*	not shiftable	not stressed
B	verb + PP-object *(*e.g.*, agree with someone)*	not shiftable	unstressed
C	verb + part + NP *(*e.g.*, look up the word)*	shiftable	stressed

The table illustrates a gradient from verb–PP constructions with low verb–particle integration (Types A and B) to phrasal verb constructions with higher degrees of lexical cohesion (Type C), a distinction that motivates the experimental contrast examined in this study. The distinction between *unstressed* and *not stressed* is intended to capture different prosodic properties: *unstressed* refers to the absence of prosodic prominence under neutral sentence stress, whereas *not stressed* refers to resistance to contrastive or focal stress.

When determining the lexical status of verb–particle combinations - for example, whether it is appropriate to regard a particular verb–particle combination as a unit in the lexicon - the factor of meaning plays an essential role, particularly the degree to which the combination’s meaning is predictable from its parts (Cruse, 1986; Cowie and Mackin, 1975). Combinations with non-compositional or idiomatic meanings (e.g., give up meaning ‘quit’) are often assumed to be more likely candidates for lexical storage than semantically transparent combinations.

It is not surprising that phrasal verbs were treated as a special type of verbs in many approaches (Fraser, 1976; Fraser, 1966; Fraser, 1965; Görlach, 2004), also, Lipka (1972), who stated that this category of verbs “can be regarded as a particular surface structure shared by a large number of lexical items with various word-formative and semantic structures” (13–17). However, it must be underscored that this does not mean that phrasal verbs are a clearly defined category in that, as the lexicographical description provided by Cowie and Mackin (1993) in the Oxford Dictionary of Current Idiomatic English (ODCIE) has shown, by no means all members of the category share all of its features.

The key issue that we wish to address here is whether there is any cognitive and neurolinguistic evidence to support one of the claims (as identified in Quirk et al., 1985, p. 1156) that prepositional verbs show similar processing patterns to phrasal verbs, i.e., whether they might be represented as unified lexical items or whether, as argued in valency theory and in Huddleston and Pullum (2002), prepositional phrases are best treated as syntactic complements of the verb in very much the same way that noun phrases, that-clauses or to-infinitives are.

### 1.2 Verb-particle combinations in experimental studies

Eye-tracking studies directed to verb-particle combinations have emerged as a research method in both linguistics and psycholinguistics. These studies, by examining the processing and comprehension of verb-particle combinations, shed light on the cognitive processes regarding language production and comprehension. Herbay et al. (2018) examined how French-English bilinguals process Verb-Particle Constructions (VPCs) specifically phrasal verbs within sentences, drawing on a framework proposed by Gonnerman and Hayes (2005). They investigated whether bilingual processing parallels that of monolinguals, emphasizing the particle position, NP length, and phrasal verb dependency. Their findings clarified the influence of phrasal verb lexical knowledge and working memory on reading patterns. Bilinguals with limited English phrasal verb comprehension showed limited processing, whereas those more proficient in English phrasal verbs displayed native-like reading behaviors during phrasal verb comprehension. This study investigated the significance of phrasal verb lexical knowledge in sentence processing since deficits in this knowledge can hinder syntactic processing in second-language sentence understanding.

In another study, Tiv et al. (2019) explored the comprehension of verb-particle combinations, specifically phrasal verbs, among adults during L1 and L2 Reading, in which they considered these combinations as a domain recognized for their inherent challenges for second language (L2) learners. The results showed that there are three factors influence phrasal verbs processing: form, semantic transparency, and frequency. Where Monolingual studies show that transparent phrasal verbs (e.g., “*pick up*”) are processed more easily than opaque ones (e.g., “*make up*”). Frequent VPCs are generally processed more quickly, but form and transparency modulate these effects. Moreover, L1 English readers did not present a clear preference for either the adjacent or split particle positions. This observation indicated that other item-specific attributes, such as frequency and co-occurrence strength, possibly modulate reading patterns.

In contrast, L2 speakers often avoid VPCs in production, preferring single-word alternatives, especially for opaque forms. The comprehension studies indicate that L2 speakers process phrasal verbs differently from L1 speakers, where early L2 demonstrating faster reading times than late L2. Preferences for adjacent forms appear influenced by language pair similarities and working memory constraints. The findings of the study indicate that L2 phrasal verb reading is modulated by familiarity with English usage, with individual differences that shape the processing patterns.

In the context of the neurolinguistic experiments that suggest phrasal verbs are processed as single lexical units in the brain (Cappelle et al., 2010), further discussed by Pulvermüller et al. (2013, p. 415). The study showed that phrasal verbs are processed as single lexical units rather than as separate syntactic components. This effect was observed regardless of whether the combination had a literal or idiomatic meaning, suggesting a unified cortical memory circuit for phrasal verbs. The results support the hypothesis that the link between the verb and its particle is lexical in nature, as evidenced by the Mismatch Negativity (MMN) responses (Näätänen, 1995; Näätänen et al., 2007) to valid combinations like “*rise up”* and “*heat up.”*

The processing of phrasal verbs, whether attributed to lexical storage, frequency, or predictability might align with broader psycholinguistic evidence. Arnon and Snider (2010) showed that even compositional multi-word units are processed more efficiently when they are frequent, suggesting that high transitional probabilities may facilitate holistic retrieval. On the other hand, linguistic constructions, with their lower co-occurrence frequencies and syntactic flexibility, likely require progressive integration. This distinction is further supported by Smith and Levy (2013) finding that word predictability follows a logarithmic relationship with reading times: highly predictable constructions are processed faster, while less predictable sequences demand greater cognitive effort. These studies highlights the interaction between usage-based factors such as frequency and predictability and real-time processing, motivating our investigation of how frequency, predictability, and construction type jointly shape eye-movement behavior.

The broader three-category classification schemes introduced above serve to situate the theoretical landscape of verb-particle constructions. The present study, however, focuses on a reduced two category contrast, which are phrasal verbs and verb-preposition combinations (as called by Quirk et al., 1985: prepositional verb), because these constitute the two theoretically distinct components that are jointly realized in phrasal-prepositional verbs. Since phrasal-prepositional verbs presuppose this distinction, the most informative experimental question is whether these two component construction types themselves differ in real-time processing. Accordingly, the present study targets phrasal verbs and verb-preposition combinations directly and excludes phrasal-prepositional verbs from the experimental design.

### 1.3 This study

Given the theoretical debate outlined above, these two construction types constitute the minimal and most informative experimental contrast for testing lexical-unit versus compositional processing accounts. The key motivation of this study is to test whether the valency theory’s predictions about distinct processing patterns for phrasal verbs and verb-preposition combinations hold true in real-time reading. While prior eye-tracking and neuroimaging research on verb–particle combinations in English have focused primarily on phrasal verbs (e.g., Cappelle et al., 2010; Tiv et al., 2019; Herbay et al., 2018), little attention has been paid to their direct comparison with verb-preposition combinations. Recognizing this gap, the present study comparatively assesses the cognitive processing associated with phrasal verbs and verb–preposition combinations during English L1 reading, using eye-tracking measures to examine early and late stages of processing.

## 2 Methods

### 2.1 Original dataset (Provo corpus)

In this study, we use Provo eye-tracking Corpus (Luke and Christianson, 2018). The Provo corpus consist of 55 short passages that were sourced from various materials like news articles, science magazines and fiction, averaging 50 words in length (range: 39–62) with 2.5 sentences each (range: 1–5). Each sentence had about 13.3 words (range: 3–52). In total, the passages had 2,689 words, with 1,197 unique word forms. The eye-tracking data that this study used was extracted from the previously mentioned corpus, which was collected from an experiment that recruited native American English speakers (*n* = 84), who had 20/20 corrected or uncorrected vision. Participants were presented with the written passages. The passages were presented one at a time on a computer screen, with eye-tracking technology used to measure the participants’ eye movements when they read the sentences.

The data were collected with an SR Research EyeLink 1,000 plus eye-tracker sampled at 1000 Hz (for the full description of the method and stimuli, see Luke and Christianson, 2018). This corpus was ideal for our purpose as it contained a reasonable number of verb-particle combinations with variant categories, and the eye movement measures were provided for all the words in the corpus.

### 2.2 Extracted regions of interest

In the context of our investigation, we directed our focus toward phrasal verbs and verb-preposition combinations, which in regular language use are often used in more complex syntactic structures. To make the target words analysis simple, we extracted these specific types of verb-particle constructions from the larger corpus. This enabled the analysis to concentrate on the fundamental lexical verbs and the particles involved in these constructions’ formation. The extracted dataset consists of a total of 19 VPCs, which were further categorized into two subsets: 10 phrasal verbs and 9 verb-preposition combinations. The phrasal verbs in the corpus were comprised of lexical elements tagged as “Verb” paired with adverbial particles tagged as “Adverb.” In parallel, the verb-preposition combinations included in our study were characterized by lexical verbs tagged as “Verb” which were conjoined with prepositional particles annotated as “Preposition.” The VPCs also varied in other aspects (e.g., verb tense), which were not directly relevant to the current study. Thus, only the lexical verb itself was taken into account, particularly concerning lexical factors such as word length, while auxiliary verbs and other surrounding elements that could affect word length in actual usage were excluded. For instance, while a sequence like “was taking off” is longer than “depend on,” our analysis considered only the verb tokens (“taking” vs. “depend”), ensuring that auxiliary verbs did not influence the length count.

### 2.3 Eye tracking measures

Eye tracking systems record several measures that represent the eye reactions when dealing with a specific word or region of interest. These measures are separated into two groups, early and late measures. Early measures of reading are posited to capture preliminary, pre-semantic stages of lexical access within memory, specifically focusing on the retrieval of a word’s form devoid of semantic representation. In contrast, late measures are theorized to reflect subsequent semantic processes, including the understanding of a lexical item and its contextual integration within the context (Haeuser et al., 2021).

In this work, we focus on three early measures: First fixation duration (The duration of the first fixation on the interest area, in milliseconds), which primarily stands for lexical information processing, like lexical access (Inhoff, 1984) and is usually affected by lexical factors such as length, frequency and predictability (Staub and Rayner, 2007; Carrol and Conklin, 2015). Gaze duration/first-pass reading time—the sum of fixation durations during the first pass within a lexical verb. This measure reflects lexical access, as it captures the total time spent on the word before the reader moves to the next region of interest (Inhoff & Radach, 1998; Rayner, 1998; Salicchi et al., 2023). Go-past time, the total fixation duration from when the reader first fixates on a target word, including any regressions to earlier words in the sentence before moving forward. This measure is applied to the particle (whether adverbial or prepositional; Haeuser et al., 2021). For late measures, we consider total reading time, which is the sum of all fixation durations on both the lexical verb and the following particle, analyzed separately. This measure reflects more strategic, controlled processes involved in reading comprehension and is influenced by both lexical access efficiency and sentence-level processing, indicating a semantic representation of the word within the sentence context (Radach and Kennedy, 2013; Salicchi et al., 2023).

Early measures primarily reflect lexical access and early-stage word processing, which are largely driven by lexical properties such as frequency, predictability, and length. However, they are not entirely devoid of semantic influences, as prior research suggests that some degree of semantic processing can occur during early fixation stages (Staub and Rayner, 2007; Carrol and Conklin, 2015). In contrast, late measures, such as total reading time, are associated with more controlled, integrative processes that involve sentence-level comprehension, reflecting a more sustained engagement with semantic and syntactic structures (Radach and Kennedy, 2013; Salicchi et al., 2023).

### 2.4 Length, frequency, and predictability testing

While there are various English Corpora for linguistic pattern research, we used the Corpus of Contemporary American English (COCA) for the frequency analysis considering that the eye-tracking data is collected from native American English speakers. Thus, other corpora like the British national corpus would not be the perfect resource to define the correlations between the word frequencies and eye-tracking measures. The analysis investigated the frequency of verb, verb-particle combinations, and individual particles.

Regarding predictability, there is a growing interest in using the large language models’ capabilities to assess the linguistic processing and predictabilities (Hosseini et al., 2024) through tasks like next-word prediction with recurrent neural networks such as LSTM (Long Short-Term Memory; Hochreiter and Schmidhuber, 1997), and fill-in the blank task with pre-trained language models such as BERT (Bidirectional Encoder Representations from Transformers; Devlin et al., 2019). Following Smith and Levy (2013) finding that word predictability logarithmically affects reading times, we used BERT to quantify the predictability of target verbs in their contexts. This method has been used to distinguish the construction-specific processing costs from general predictability effects. We used the original sentences presented to participants in the eye-tracking experiment and systematically masked the target verbs. We then provided these masked sentences to BERT and extracted the model’s predicted probabilities for the target verbs.

**Figure 1:**
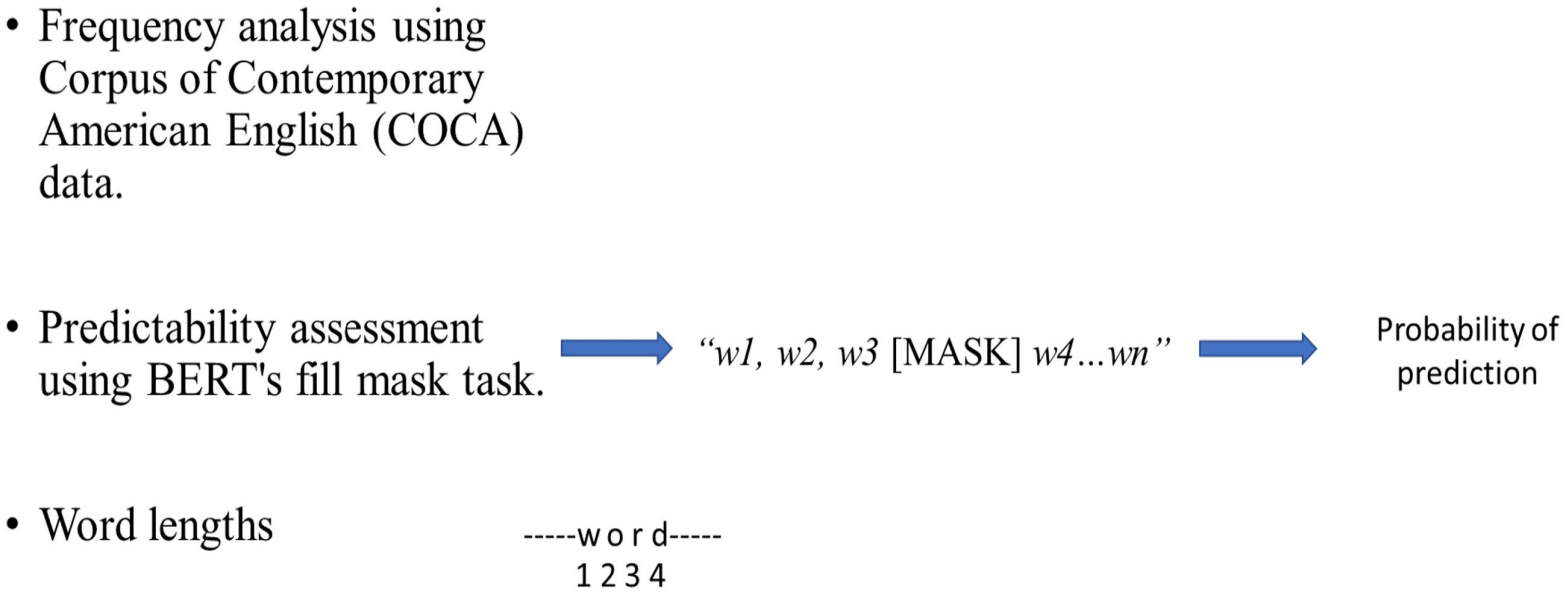
The procedures to define frequency, predictabilities, and lengths of VPCs as usage-based information for analyzed constructions.

In addition to frequency and predictability, we also considered the length of each verb-particle combination, measured by the number of characters in the verb sequence (e.g., *depend* in *depend on* = 6 characters, *look* in *look up* = 4 characters). This allowed us to test the potential effects of orthographic length on reading time (Figure 1).

### 2.5 Statistical testing

For our statistical analyses, we first tested the normal distribution of data for both the eye-tracking measures and usage-based factors using the Kolmogorov–Smirnov test, which reported significant deviations from normality across all assessed measures and factors. The statistics and corresponding *p*-values indicated substantial non-normality (see Table 2). Thus, we used a non-parametric method for hypothesis testing. We computed separate Mann–Whitney U tests (Mann and Whitney, 1947; McKnight and Najab, 2010) for both the eye-tracking measures and usage-based factors, each variable is being computed with respect to the category of the VPCs (PV: phrasal verb and PRP: verb-preposition combination). Therefore, we run a non-parametric correlation analysis with Spearman correlation to detect any potential correlation between the eye movement patterns and the usage-based information of the defined VPC. All the statistics are implemented with Python, and NumPy (Harris et al., 2020).

**Table 2:** Kolmogorov–Smirnov test for normality of eye-tracking measures and usage-based factors.

Measure/factor		Statistics	*p*
Eye tracking measures	First fixation duration	0.11	<0.001
	Gaze duration	0.14	<0.001
	Go-past times	0.28	<0.001
	Total reading time (verbs)	0.17	<0.001
	Total reading time (particles)	0.19	<0.001
Usage-based factors	Length	0.36	<0.001
	Frequency	0.22	<0.001
	Predictability	0.21	<0.001

To address the language-as-fixed-effect concern (Clark, 1973) and account for the non-independence of eye-tracking observations across both participants and lexical items, we fitted linear mixed-effects models with crossed random intercepts for participants and items. For each eye-tracking measure, the dependent variable was log-transformed [log (1 + duration)] to reduce skewness. Construction type (phrasal verb vs. verb–preposition combination) was entered as a fixed effect. This approach allows inference to generalize simultaneously over subjects and stimuli within a single model and is now standard practice in psycholinguistic research.

No outliers were removed from the eye-tracking data prior to analysis. This decision was motivated by the use of a well-established, preprocessed corpus (the Provo Corpus) and by the robustness of the statistical models employed. As a robustness check, all mixed-effects analyses were repeated after excluding extreme values (>3 SD from the mean).

## 3 Results

### 3.1 Eye-tracking results

The results showed that the reading times, as represented in the first fixation duration, did not differ between the two verb types, the median of the first fixation duration for both categories were very similar, with 200 ms for phrasal verbs and 194 ms for verb-preposition combinations. The Mann–Whitney U test (U = 180567.5; *p* = 0.543) reported no difference between the two categories with respect to the dependent variable, first fixation duration.

Similarly, gaze duration did not show a reliable difference between construction types: participants spent approximately the same amount of time on phrasal-verb tokens as on verb-preposition-combination tokens. Median gaze duration was 208 ms for phrasal verbs and 207 ms for verb-preposition combinations. The Mann–Whitney U test did not detect a reliable difference (U = 171,549; *p* = 0.361).

The median go-past times for particles were slightly different (numerically). 191 ms for phrasal verbs and 200 ms for verb-preposition combinations. Nevertheless, they did not show a statistically significant difference (U = 75,445; *p* = 0.178).

Across early measures (first fixation duration, gaze duration, and go-past time), no reliable differences were detected between phrasal verbs and verb–preposition combinations. However, given the limited and heterogeneous item sample, these null results should be interpreted with caution and cannot be taken as evidence for equivalent processing.

Total reading time, a late measure associated with integrative processing, also did not show a reliable difference for lexical verbs: median total reading time was 219 ms for phrasal verbs and 223 ms for verb–preposition combinations (U = 171,765; *p* = 0.354).

**Figure 2:**
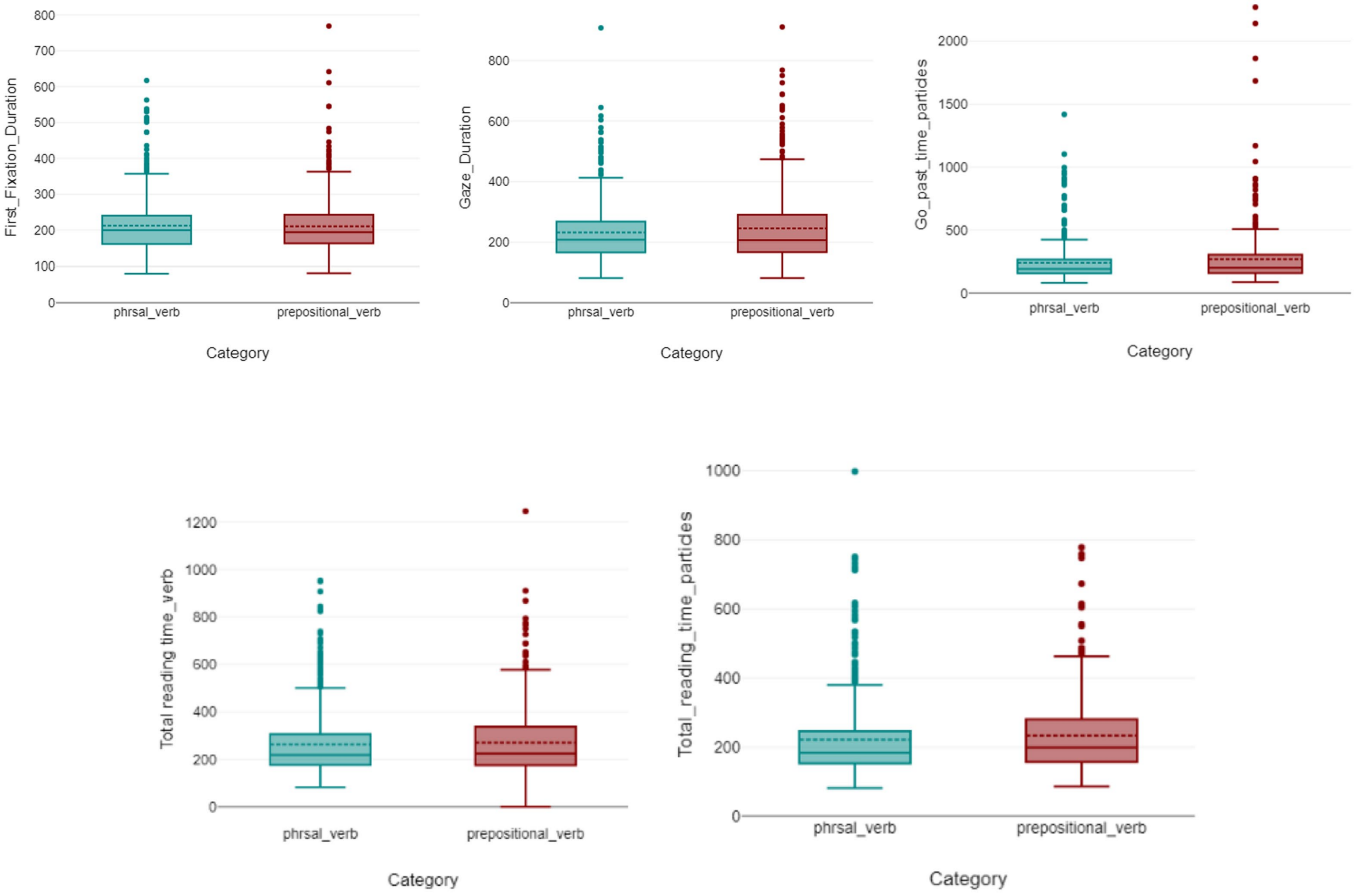
Visualizations of first fixation duration, gaze duration, go-past times, and total reading times for lexical verbs and particles in phrasal verbs and verb-preposition combinations. In each boxplot, the horizontal line indicates the median, while the dot represents the mean of the distribution. The box spans the interquartile range (25th–75th percentiles); whiskers extend to the most extreme values within 1.5 × IQR, and points beyond the whiskers represent outliers.

No reliable differences were detected between conditions. However, given the limited number of items and the resulting power constraints, this null result cannot be taken as evidence for equivalent processing (Figure 2).

However, the total reading times of particles showed significant differences, participants showed a different efficiency reading phrasal verbs in times less than verb-preposition combinations. The median for particles in phrasal verbs was 184 ms while for verb-preposition combinations was 198 ms. Therefore, the total reading time measure showed a statistically significant difference between the particles in the two distinct categories (U = 72563.5; *p* = 0.0168). These observation-level comparisons are complemented by mixed-effects analyses that explicitly account for participant and item variability (see Section 3.3).

### 3.2 Length, frequency and predictability as usage-based information for the defined VPC

These results, while indicating some notable cognitive and linguistic similarities in the processing of phrasal verbs and verb-preposition combinations, may also be influenced by external factors, such as the length, frequency and predictability of the verb constructions. This is what the second question addresses. Therefore, we test another hypothesis on the equality of the two groups in terms of these external factors.

**Figure 3:**
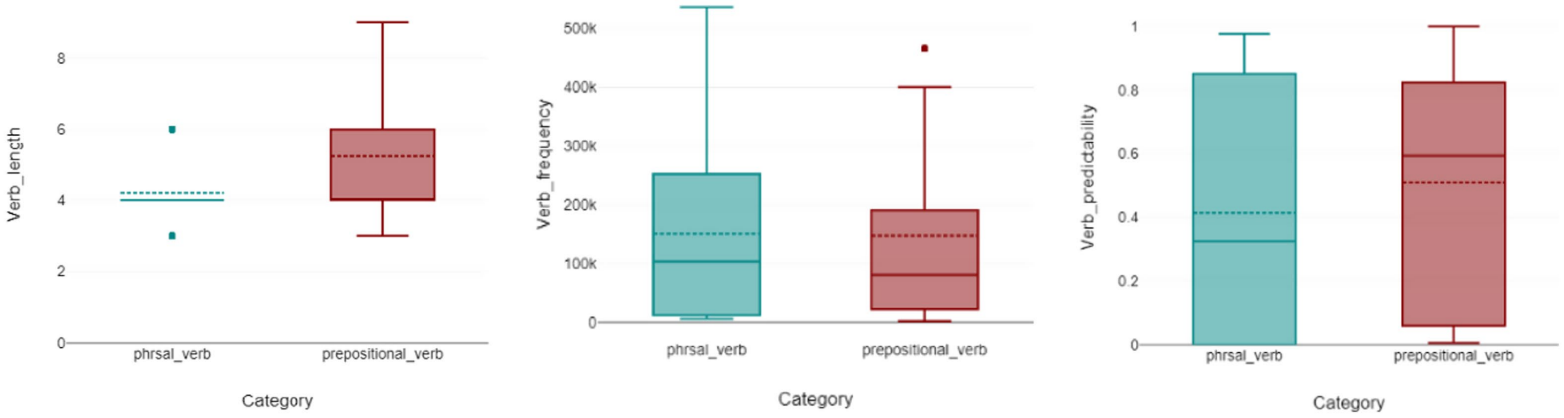
Boxplots showing usage-based properties of phrasal and prepositional verbs. Left: Token length of each verb construction type; Center: Frequency (from corpus counts); Right: Predictability scores. Significant differences were observed for length and predictability (*p* < 0.001), but not for frequency (*p* = 1.0). In each boxplot, the box spans the interquartile range (Q1–Q3) with the median as the horizontal line; whiskers extend to the most extreme values within 1.5 × IQR, and points beyond the whiskers represent outliers.

The statistical analysis of length indicated a statistically significant difference between the two types of constructions (U = 218,736; *p* < 0.001), suggesting that there is a significant variation in the length of phrasal verbs compared to verb-preposition combinations. Phrasal verbs had a mean length of 4.2 and a median of 4, whereas prepositional verbs had a mean of 5.2222 and the same median of 4. The results of the predictability test also showed a statistically significant difference between the two verb types (U = 232,848; *p* < 0.001), indicating that there is a discernible variation in predictability between phrasal and verb-preposition combinations. Phrasal verbs had a mean predictability score of 0.414 and a median of 0.3254, while prepositional verbs showed a higher mean of 0.5093 and a median of 0.5937. However, the frequency of the two categories of these constructions did not demonstrate a significant difference, as evidenced by (U = 317,520; *p* = 1), even though the phrasal verb group had higher values for frequency (Mean = 150844.4; Median = 103,459) than the prepositional verb group (Mean = 147623.5556; Median = 80,739; Figure 3).

The most remarkable correlation between the usage-based information and eye movement measures is observed in the length of the verb construction with total reading time, where the highest significant positive correlation has been reported in phrasal verbs with an (*r* = 0.21, *p* = 0.001). A negative correlation is noted in phrasal verbs between the higher predictability that correlates with the short total reading times by (*r* = −0.19, *p* < 0.001). However, there was no correlation between the verb in verb-preposition combinations category and this eye movement measure (Table 3).

**Table 3:** Spearman correlation analysis.

Feature	Construction	First fixation duration		Gaze duration		Total reading Time_verbs	
		*r*	*p*	*r*	*p*	*r*	*p*
Length	PV	0.03	0.448	0.09	0.024	0.17	<0.001
	PRP	−0.03	0.528	0.13	0.001	0.11	0.01
Frequency	PV	−0.01	0.828	−0.03	0.453	−0.09	0.025
	PRP	−0.04	0.29	−0.17	0.001	−0.18	<0.001
Predictability	PV	−0.06	0.142	−0.1	0.015	−0.19	<0.001
	PRP	0.04	0.305	0.01	0.876	−0.01	0.726

### 3.3 Mixed-effects analysis

Linear mixed-effects models with crossed random intercepts for participants and items revealed no reliable differences between phrasal verbs and verb–preposition combinations for early verb-region measures, including first fixation duration (*p* = 0.412) and gaze duration (*p* = 0.161), nor for total reading time on the verb (*p* = 0.593). Regression path duration on the particle region showed a non-significant trend (*p* = 0.062), which did not remain after excluding extreme values (*p* = 0.229). In contrast, total reading time on the particle region was reliably lower for phrasal verbs than for verb–preposition combinations (*β* = −0.088, *p* = 0.0108). This effect remained robust after excluding extreme values (>3 SD; *β* = −0.099, *p* = 0.0027).

## 4 Discussion

This study examined the cognitive processing of verb–particle constructions during continuous sentence reading using eye-tracking measures. Within a valency-theoretic framework, we investigated how native English speakers process sequences of verb + particle + NP, focusing on differences between phrasal verbs and verb-preposition combinations. Eye-movement patterns provided insight into the dynamics of reading and the underlying cognitive mechanisms involved in processing these constructions. In addition, we examined how usage-based factors-specifically length, frequency, and predictability-modulate eye-movement behavior and interact with construction type.

### 4.1 Generalization over linguistic items

A central methodological concern in psycholinguistic research is whether results generalize beyond the specific stimuli tested (Clark, 1973). To address this issue, we applied linear mixed-effects models with crossed random effects for participants and lexical items, enabling generalization over both sources of variability. These analyses showed a reliable difference between phrasal verbs and verb-preposition combinations only for particle-level total reading time, whereas earlier measures and verb-region measures did not differ. This pattern supports an interpretation in terms of differential lexical integration at the particle level, while warranting caution in extending the findings to broader syntactic categories given the limited number of construction exemplars in the corpus.

### 4.2 Phrasal verbs versus verb-preposition combinations

#### 4.2.1 Predictability and efficient lexical access in phrasal verbs

The results reveal a negative correlation between predictability and eye-tracking measures for phrasal verbs: more predictable phrasal verbs are associated with shorter total reading times. This pattern suggests that highly predictable phrasal verbs are accessed and processed more efficiently during reading. Such an effect is consistent with linguistic and neurolinguistic accounts proposing that phrasal verbs function as single lexical units (Cappelle et al., 2010; Pulvermüller et al., 2013).

#### 4.2.2 No clear predictability effects in verb–preposition combinations

In contrast, predictability did not reliably correlate with eye-tracking measures for verb–preposition combinations. This absence of a predictability effect suggests that visual processing efficiency for these constructions is less strongly shaped by usage-based expectations, pointing to differences in how predictability contributes to the processing of distinct verb-particle constructions.

#### 4.2.3 Connection with past research

Our study aimed to contribute to the ongoing discourse surrounding the processing of verb-particle combinations in the two distinctions, phrasal verbs and verb-preposition combinations. Traditional grammar models (Quirk et al., 1985) often treat both phrasal and verb-preposition combinations as single lexical units, exemplified in sentences like “She looked after her son”, where the sequence “looked after” is considered a single lexical unit called prepositional verb. However, the valency theory interpretation, proposed by Herbst and Schüller (2008), introduces an alternative perspective on analyzing these combinations. In this approach, valency relates to the number and types of arguments that a predicate, mainly a verb, can govern. Considering verb-preposition combinations like “decide on”, “decide against”, and “decide in favour of”, the valency theory provides an alternative interpretation, instead of viewing these combinations as single lexical units, as some grammatical frameworks might (Quirk et al., 1985), valency theory perceives the main verb “decide” and its prepositional complements such as “on”, “against”, “in favour of” as separate yet fundamentally interconnected elements that contribute to the predicate’s valency. For instance, in the sentence:

- They decided on the bus.

The verb “decide” is considered as the predicator. Meanwhile, the prepositional phrase “on the bus” is identified as one of the potential complement classes fulfilling the verb’s valency requirements. This interpretation offers multiple benefits, such as consistent formalization across various usages of the verb and its complements. Crucially, it avoids unnecessary expansion of the number of lexical units in consideration, enabling a systematic description based on the semantic roles of different complement classes. This perspective supports the comprehension of the intricate syntactic relationships between the verb, its prepositional complement, and other sentence components.

Within the valency-theoretic framework adopted here, our findings do not provide strong evidence for distinct processing patterns between phrasal verbs and verb-preposition combinations in early eye-tracking measures. A difference emerged only in a late measure, namely total reading time on the particle. While neurolinguistic studies have suggested a distinct neural processing mechanism for phrasal verbs, our behavioral data differentiate the two construction types only at the level of particle processing, which is typically associated with later, semantically integrative stages of comprehension. In this respect, valency theory, which predicts a more segmented processing of verb-preposition combinations, receives partial empirical support from the present results.

These results do not support a strict application of valency theory across all stages of reading, as no reliable differences between phrasal verbs and verb-preposition combinations emerged in early measures for either lexical verbs or particles, nor in late measures for lexical verbs. However, a clear difference was observed in a late measure at the particle level: particles in verb-preposition combinations elicited longer total reading times than those in phrasal verbs. This effect suggests that particles in phrasal verbs are more tightly integrated with the verb, consistent with their treatment as part of a single lexical unit and therefore attract less processing effort during reading. This interpretation aligns with experimental evidence supporting the lexical unity of phrasal verbs (Cappelle et al., 2010). In contrast, the distinct functional roles of the verb and preposition in verb–preposition combinations may necessitate additional processing, resulting in longer reading times on the particle.

## 5 Conclusion

This study contributes to the ongoing discussion on the processing of verb-particle combinations by highlighting the difficulty of mapping theoretical linguistic distinctions, such as those proposed by valency theory, directly onto behavioral measures of real-time reading. Although valency theory posits a clear linguistic and cognitive distinction between phrasal verbs and verb–preposition combinations, our findings indicate that this distinction is not uniformly reflected across eye-tracking measures during natural reading.

Future research is needed to further clarify the relationship between theoretical linguistic models and cognitive processing mechanisms. In particular, studies incorporating additional linguistic features and experimental manipulations may help determine the conditions under which theoretical distinctions emerge behaviorally. Beyond proficiency-related effects, future work could also examine how different semantic states of verb–particle constructions (literal, semi-idiomatic, and fully idiomatic) influence processing. While the present study investigated these constructions in natural sentence contexts, an open question remains whether the preference for figurative interpretations persists when sentence or discourse contexts bias readers toward literal meanings.

More broadly, the present findings provide a foundation for future work at the intersection of network neuroscience and computational modeling of language. The differential emergence of processing effects at the particle level places constraints on how verb-particle constructions may be represented within distributed neural and computational networks, suggesting graded rather than categorical differences in lexical integration. These constraints are directly relevant for network-based models of language processing, including connectionist and transformer-based architectures, which aim to capture both compositional structure and usage-based regularities. Integrating eye-tracking data with representational analyses from computational models or with network-level measures derived from neuroimaging may help clarify how multi-word expressions are encoded across levels of processing, thereby contributing to a more mechanistic, neurocognitively grounded account of linguistic structure.

A limitation of the present study concerns the restricted and heterogeneous set of linguistic items. The analyses were based on 10 phrasal verbs and 9 verb-preposition combinations, which vary with respect to tense and lexical properties. As a result, the present findings should not be interpreted as general claims about the processing of phrasal verbs or verb-preposition constructions as grammatical classes. Rather, they reflect processing patterns observed for this specific and limited sample of items.

Given the small number of items, the study is likely underpowered to detect small or medium-sized effects at the item level. Consequently, the generalizability of the results is limited, and caution is required when interpreting both significant and non-significant effects.

Importantly, the present study should be regarded as exploratory in nature. Its primary contribution lies in illustrating how fine-grained eye-tracking measures can be applied to the study of multiword verb constructions during reading, rather than in providing a definitive test of theoretical accounts of phrasal verb processing. Future studies with larger and more systematically controlled item sets will be required to assess the generality of the observed patterns.

## Data Availability

Publicly available datasets were analyzed in this study. This data can be found at: Luke and Christianson (2018).

## Footnotes

1 In this study, we use the term “particle” as a cover term for both adverbial and prepositional elements that combine with verbs (cf. Quirk et al., 1985, p. 1160; Huddleston and Pullum, 2002, p. 274). While some frameworks reserve “particle” for adverbial elements only, we adopt a broader usage for consistency across verb-particle constructions (VPCs).

2 From the cited grammar: “Phrasal-prepositional verbs: There is a further major category of multi-word verbs which will be called *phrasal prepositional verbs*, because they contain, in addition to the lexical verb, both an adverb and a preposition as particles” (Quirk et al., 1985, p. 1160).
